# A Skill Acquisition Perspective on the Impact of Exergaming Technology on Foundational Movement Skill Development in Children 3–12 Years: A Systematic Review and Meta-analysis

**DOI:** 10.1186/s40798-022-00534-8

**Published:** 2022-12-16

**Authors:** Luca Oppici, Frederike Marie Stell, Till Utesch, Carl T. Woods, Lawrence Foweather, James R. Rudd

**Affiliations:** 1 The Department of Teacher Education and Outdoor Studies, Norwegian School of Sport Sciences, Oslo, Norway; 2 Institute of Communication and Media Research, German Sport University Cologne, Cologne, Germany; 3 Institute of Educational Sciences, University of Münster, Münster, Germany; 4 Institute for Health and Sport (IHES), Victoria University, Melbourne, Australia; 5 Research Institute for Sport and Exercise Sciences, Liverpool John Moores University, Liverpool, UK; 6 Western Norway University of Applied Sciences, Sogndal, Norway

**Keywords:** Skill acquisition, Fundamental movement skills, Technology, Exergaming, Motor competence, Foundational movement skills

## Abstract

**Background:**

Sedentary, digital screen time in children represents a major concern due to its detrimental effect on children’s development. Nowadays, however, advances in technology allow children to actively interact with a digital screen using their whole body (e.g., exergaming), providing potential for movement learning. Exergaming technology may prove valuable in supporting children’s development of foundational movement skills (FMS).

**Objective:**

To examine the impact of exergaming technology on the development of FMS in children 3–12 years through a skill acquisition lens.

**Methods:**

Systematic review and meta-analysis were conducted following the PRISMA guidelines. Web of Science, PubMed, PsycINFO and SPORTDiscus databases were searched between 2007 and 2022. Studies were eligible if they conducted an exergaming intervention to improve FMS in typically developing children aged three to twelve with a control group, using a baseline and post-intervention assessment design. FMS outcomes were pooled with a random effects model.

**Results:**

Nine trials (4 RCTs, 2 cluster RCTs and 3 non-randomized trials) of varying methodological quality (2 had low, 6 had some concerns, and 1 had a high risk of bias) were included, with a total of 783 participants. FMS outcome measures across studies comprised object control skills, locomotor skills, coordination, agility, balance and balance-related skills. The meta-analysis included showed a small positive effect in favor of the exergaming intervention (*r* = 0.24 [95% confidence interval: 0.11–0.36]).

**Conclusion:**

Our results indicate that screen-based technology that requires an active engagement of the child can promote the development of FMS. Considering that FMS are the foundation of a child’s physical, mental, health and academic development, this finding could lead to a reshaping of the perception of digital screen-based technology and the role this should play in children’s lives. We speculate that the observed benefits most likely depend upon the quality of information–movement coupling specificity and the motor learning strategies built into the exergame and/or the intervention design. We do not believe this is dependent on the type of FMS being performed or the amount of practice. We recommend therefore that future research should examine how practitioners (school teachers, coaches and parents) can facilitate the interaction between a child and exergaming technology.

## Key Points

- Screen-based technology is typically considered negative for children, as it promotes sedentary behavior. Contrarily, screen-based technology that requires a whole-body active engagement of the user may encourage the development of foundational movement skills (FMS).
- Our meta-analysis showed a small effect (*r* = 0.24) of practicing exergaming technology for developing FMS in children age 3–12.
- Practitioners (teachers and coaches) can use this technology to supplement their lessons (e.g., in home-based practice). We provide guidelines for an effective implementation and development of such technology.

## Introduction

Children are the fastest growing users of digital technology [1, 2], with those up to the age of 8 years being estimated to use on average digital screens for approximately 25% of their waking hours [3]. Typically, children engage in screen time in a sedentary manner, passively digesting digital information with minimal body movement when watching digital media or playing digital games (e.g., pressing buttons). It has been widely shown that children’s digital screen time while sedentary is a cause of concern for global health, as passive screen use in children is associated with negative consequences for their physical, mental and social development [4–9]. These findings have led to recommendations by academics and governments to limit children’s use of screen time technologies. For example, it is suggested that children under the age of five should have no more than 60 min exposure a day; however, concerns have been raised that two out of three children are currently not meeting these guidelines [3]. Indeed, the COVID-19 pandemic has highlighted how difficult this target is to achieve, as screen-based technology was primarily used for conducting home-based school lessons to facilitate social distancing [10].

A skill acquisition perspective views children’s screen time based on how children interact with screen-based technology. This results in a shift away from the current perspective of seeing screen time as being bad per se, instead moving toward a perspective of investigating the interactive *relationship* between the user (child) and the screen. Advances in technology allow children to actively interact with a digital screen using their body (not only by pressing a button), providing potential for movement learning. In this systematic review, we explore—from a skill acquisition perspective—how practicing whole- or part-body movement in response to a variety of stimuli from a screen (immersive and interactive technology) can influence the development of Foundational movement skills (FMS). These technologies offer new opportunities for digital engagement, potentially even facilitating children’s FMS development rather than diminishing it. We believe that considering the issue of screen time use in children from a skill acquisition perspective may further advance our understanding, helping academics and governments to find new ways to tackle this global problem.

Foundational movement skills are defined as goal-directed movement patterns that directly and indirectly impact an individual’s capability to be physically active and can be developed to enhance physical activity participation and promote health across the lifespan [11]. Supporting FMS is a worthy developmental and educational aim as FMS represent a critical aspect of children’s development, and it enhances a child’s capacity to participate meaningfully in play, games and activities, and is a key enabler of children’s physical activity throughout the life course [12, 13]. The development of FMS is also associated with positive trajectories of academic achievement, mental health and quality of life [14]. Traditionally, FMS are largely developed in physical education (PE) classes, such as catching and throwing, and support a child’s ability to perform in a variety of contexts (e.g., at the playground with their friends) and sports (e.g., cricket and basketball). To support the acquisition of FMS, the principle of specificity of practice [15] is of paramount importance and commonplace in PE teaching. The principle of specificity means the coupling between information and movement is critical, in the sense that children should learn to regulate their movement on information specific to intended scenarios [16]. For example, for improving tennis striking skills, a child should practice hitting a moving tennis ball with a tennis racquet. Through interaction with a wide range of activities that involve throwing and catching—e.g., basketball, rugby, cricket, baseball—children progressively become more attuned to specifying information that guides continued exploration of their surrounding; over time, this will lead to the development of FMS [17, 18]. Consequently, a variety of throwing and catching skills, for example, enables children to confidently function, and perform successfully, across multiple sporting and physical activity environments [19]. In fact, children with high levels of object control skills (e.g., throwing, catching, kicking) are often more active throughout childhood, in addition to being more physically active in late adolescence and beyond [12, 14, 20].

As noted, interactive and immersive screen time technology has the potential to contribute to the development of FMS in children. Interactive and immersive technologies, such as exergaming (e.g., Wii, Nintendo, Kyoto, Japan; Xbox Kinect, Microsoft, Redmond, WA), were created to increase users’ movement activities, engagement and enjoyment in screen time activities. Exergaming has the potential to create an interactive environment where children explore and adapt to a wide range of movement activities—from athletics to bowling, tennis and basketball (among others). As opposed to the traditional digital pressing of buttons on a controller, children interact with exergames using movement of their body while holding a controller, whereby the movement/manipulation of the controller in space translates to the movement seen on screen. For example, while standing, a child swings their arm to perform a forehand in a tennis exergame. This exergame–child interaction involves a continuous coupling of information and action (e.g., to detect forehand opportunities for beating the opponent and actualizing this opportunity), which is the hallmark of FMS development from a contemporary motor learning perspective [21]. Importantly, there is potential for higher specificity of information–movement coupling (perceiving the coming tennis ball and moving the whole body to hit it) than pressing a button for intercepting the ball. Put simply, interactive technology has the potential to simulate activities performed in PE (and more generally in sport and play scenarios) and thus aid in the development of FMS. Alongside this, exergames include immersive features, such as creating personalized avatars, setting task difficulties and offering haptic feedback during movement – each of which supports the development of autonomous learning [22, 23]. Exergaming, therefore, has many features which could support the development of FMS [24]. Potentially, exergaming may be particularly beneficial for children with low FMS or low perception of FMS, who may find it difficult to participate in “real” activities but may feel more at ease and in a safer environment when participating in exergaming activities.

The COVID-19 outbreak highlighted that screen time technologies can be a key aspect of education when needing to facilitate social distancing [10], and it is therefore timely to explore the effectiveness of these technologies in supporting the development of children’s FMS. The aim of this systematic review is to explore the effectiveness of interactive technologies, such as exergames, for the development of FMS in typically developing children aged 3–12 years. Given the fast evolution of this technology and the associated quick turnover of applications, we decided to include studies published from 2007 (the year in which immersive technology was launched in the market) to the present (2022). Digital technology has frequently been viewed as promoting sedentary behavior in children and this review will seek to find out whether it can, to the contrary, actually promote the development of FMS, thus leading to a reshaping of the perception and role of digital screen-based technology in our society.

It is important to highlight that a systematic review with a similar question has recently been published [25]. However, we consider it necessary to conduct a new systematic review to further elucidate how interactive technology influences FMS development in children and can thus provide directions for future research. While the participants’ age is the same as for Liu et al., this systematic review focuses exclusively on FMS development (Liu et al. [25], combined physical fitness and FMS); provides a conceptualization of FMS and the related potential effect of exergaming on FMS grounded on theories of movement learning (Liu et al. [25], did not provide a theoretical consideration on how exergaming may benefit FMS); considers balance and postural-related skill as FMS aligning with current views on FMS (REF) (Liu et al. [25], considered balance only as physical ability); expands the search keywords, resulting in a higher number of studies included, relative to Liu et al. [25]; synthesizes current evidence using meta-analytic procedure (Liu et al. [25], provided a narrative synthesis which is prone to bias); and discusses results and implications on how the design of user-exergaming interaction shapes the effectiveness of an exergaming interaction (Liu et al. [25], discussed results only from a dose–response perspective). In short, this systematic review improves Liu et al.'s [25] review on theoretical and methodological aspects.

## Methods

The guidelines proposed by the 2020 Preferred Reporting Items for Systematic Reviews and Meta-Analyses (PRISMA 2020) were followed [26].

### Eligibility Criteria

PICOS statement: in 3-to-12-year-old typically developing children, does interactive and immersive technology develop FMS relative to no practice, free play or standard practice (traditional PE lessons)?

The inclusion criteria were: (a) peer-reviewed articles published as full-text in English from January 2007 to February 2022, (b) participants were typically developing children with mean age of the sample ranging from three to twelve years, (c) an intervention with immersive and interactive technology was compared to a control group (no practice, free play or standard practice), using an experimental (RCT or cluster RCT) or quasi-experimental design, and (d) the measured outcome comprised an FMS outcome (stability, locomotor and object control skills).

### Information Sources and Search Strategy

Four databases were searched to identify studies: Web of Science, PubMed, PsycINFO and SPORTDiscus. The search was last performed on the February 2, 2022. Furthermore, the references of the studies included in the review were screened for identifying extra studies.

The search strategy comprised the following syntax: (*Digital* OR Technolog* OR *Media* OR *Smartphone* OR *Tablet* OR "Video Gam*" OR *"Virtual Reality"* OR *"Augmented Reality"* OR "Mobile App*" OR Exergam* OR *eHealth* OR *Playstation* OR *Nintendo* OR *Wii* OR *Xbox* OR *Kinect* OR *Oculus* OR Wearable* OR "Mobile Device*" OR "Electronic Device*" OR *eSports* OR *"e Sports"* OR *"Fitbit"* OR *"Apple Watch"* OR *"Pokémon Go"*) AND (Child* OR Infant*) AND (Learn* OR Acqui* OR Develop* OR Train* OR Teach* OR Adapt*) AND (*Movement* OR “Motor Skill*” OR *Coordination OR “Motor Competence”*). According to the eligibility criteria, limiters “publication date” (from 1st of January 2007 to present) and “language” (English) were applied to the search strategy.

### Selection Process

**Fig. 1:**
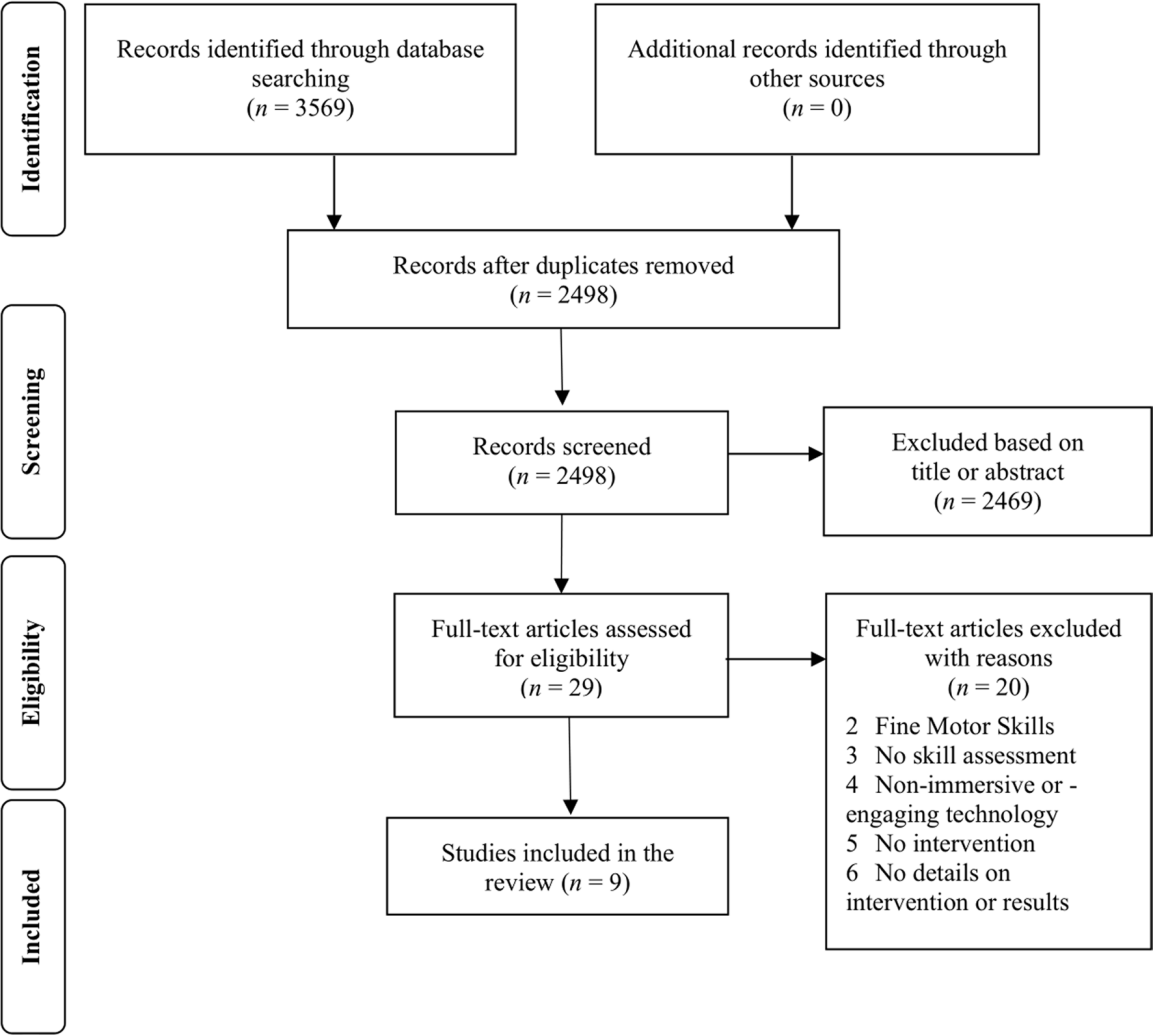
Flow diagram of the search and study selection process

The records identified through database searching were exported into Endnote X9 software (Clarivate, Philadelphia, USA), and duplicates were automatically removed. Then, two authors (LO and FS) manually screened the records excluding studies based on titles and abstracts, assessed the eligibility of the remaining full-text articles and included the studies that met the inclusion criteria (Fig. 1). The two authors independently carried out the selection process, they cross-checked their results after each step (i.e., title/abstract and full-text screening) and resolved any discrepancy in a closed meeting, and if consensus was not reached, a third author (JR) was consulted.

### Data Collection Process and Data Items

Two authors (LO and FS) manually extracted the data of interest from the included studies and compiled the data in a table format (Table 1). They worked independently, they cross-checked their results and resolved any discrepancy in a closed meeting, and if consensus was not reached, a third author (JR) was consulted.

**Table 1:** Summary of study characteristics

Reference (author, year, country)	Design and setting	Sample size, age and sex	Program duration	Device/game used in the intervention	Intervention content	FMS measures
Barnett et al. [36], Australia	Randomized controlled trial (RCT [pre–post]), after school setting	*n* = 95 6.2 ± 1.0 y 55% girls 45% boys	6 weeks, 1 day/week 60 min/session Total of 360 min	Nintendo Wii: sports active video games that require the use of object control skills Participants interacted with the software via a handheld controller	*Experimental group (EXP)*: Participants selected and played a Nintendo Wii sport game, in pairs (bowling, frisbee and/or disk golf, table tennis, tennis). A different set of games was offered each fortnight. Two research assistants supervised each session, without providing any coaching tips *Control group (CON)*: no practice	Object control skills (striking stationary ball, stationary dribble, kicking, catching, overhand throwing, underhand rolling) assessed with TGMD-2 Each item received a score of 1 (or 0) if correctly (or not) executed based on standard movement criteria
Gao et al. [34], United States	Non-randomized trial (pre–post), school setting	*n* = 65, 4.5 ± 0.5 y 51% girls 49% boys	8 weeks, 5 days/week, 20 min/session Total of 800 min	Nintendo Wii and Xbox Kinect: dance and whole-body activities (Wii Just Dance for Kids, Wii Nickelodeon Fit, Xbox Kinect Just Dance for Kids) Participants interacted with the software via a handheld controller	*EXP*: participants played the exergames individually, in pairs or in group. A teacher or research assistant supervised each session, ensuring continuous game play *CON*: participants engaged in self-selected physical activities during recess (e.g., chasing and tag)	Locomotor (running, hopping and jumping) and object control skills (throwing and kicking) assessed with TGMD-2 Each item received a score of 1 (or 0) if correctly (or not) executed based on standard movement criteria
Hsiao & Chen [41], Taiwan	Non-randomized trial (pre–post), school setting	*n* = 105, 3–6 y 47% girls 53% boys	1 day 40 min session	ASUS Xtion PRO: custom-made “goalkeeping” game Participants interacted with the software via their body movement (sensors captured children’s motion)	*EXP*: participants caught a virtual ball in different conditions *CON*: participants practiced in pairs catch and throw with the traditional instructor-led approach	Whole-body coordination (tapping a balloon to keep it in the air for as long as possible) assessed with a custom-made test
Johnson et al. [37], Australia	RCT (pre–post), school setting, lunch break	*n* = 36, 6–10 y 47% girls 53% boys	6 weeks, 1 day/week 50 min/session Total of 300 min	Xbox Kinect: sports active video games that require the use of object control skills (Kinect Sports Season 1,2; Sports Rivals) Participants interacted with the software via their body movement	*EXP*: participants chose two to three sport games to play each session in the school media room; week 1 games: tennis, table tennis, baseball; week 2 games: baseball, golf; week 3 games: table tennis, soccer; week 4 games: golf, beach volleyball; week 5 games: tennis, baseball, golf; week 6: children were given opportunity to choose their game Not specified how the sessions were supervised *CON*: no practice	Object control skills (two-hand strike, one-hand strike, ball bounce, catch, kick, underarm throw, overarm throw) assessed with TGMD-3 and a custom-made golf test Each item received a score of 1 (or 0) if correctly (or not) executed based on standard movement criteria
Mombarg et al.[33], Netherlands	RCT (pre–post), school setting, lunch break	*n* = 29 7–12 y 21% girls 79% boys	6 weeks, 3 days/week, 30 min/session Total of 540 min	Nintendo Wii: Wii Fit Plus Participants interacted with the software via a handheld controller and a balance board	*EXP*: participants chose and practiced 3 to 5 balance games in each session, for a total of 18 games throughout the intervention. The difficulty of each game was continuously and automatically adjusted A physical therapist supervised the sessions *CON*: no practice	Balance (dynamic and static) and balance-related skills assessed with M-ABC-2 and BOT-2 based on standard movement criteria
Sheehan & Katz [40], Canada	Cluster-randomized trial (pre–post), school setting, PE classes	*n* = 65 7–9 y 57% girls 43% boys	6 weeks, 3 days/week, 30 min/session Total of 540 min	Nintendo Wii: Wii Fit Plus Participants interacted with the software via a handheld controller and a balance board	*EXP*: participants practiced a list of activities on agility, balance and coordination from the Wii Fit Plus. The list of activities was of increase task difficulty and was designed to specifically improve balance. A PE specialist supervised the sessions *CON1*: participants practiced activities on agility, balance and coordination. A PE specialist ran the sessions *CON2*: participants practiced the regular PE curriculum	Balance assessed with the HUR BT4 platform
Sheehan & Katz [39], Canada	Cluster-randomized trial (pre–post), school setting, Physical education (PE) classes	*n* = 61 9–10 y 44% girls 56% boys	6 weeks, 4–5 days/week, 30 min/session Total of 720–900 min	iDance, Wii Fit Plus, XR-Board Dueller System (snowboard simulator) and Lightspace Play Wall Participants interacted with the software via a handheld controller, a balance board and sensors on the floor and the wall	*EXP*: participants rotated across the exergaming stations. Task difficulty was automatically adjusted. A PE specialist supervised the sessions *CON1*: participants practiced activities on agility, balance and coordination. A PE specialist ran the sessions *CON2*: participants practiced the regular PE curriculum	Balance assessed with the HUR BT4 platform
Vernadakis et al. [38], Greece	RCT (pre–post-retention), school setting	*n* = 66, 6.4 ± 0.7 y 45,5% girls 54,5% boys	8 weeks, 2 days/week 30 min/session Total of 480 min	Xbox Kinect; sport games that required the use of object control skills (NBA Baller Beats and Kinect Sports: Baseball mini games, NBA Baller Beats, Bowling mini games, Soccer mini games) Participants interacted with the software via their body movement	*EXP*: Xbox Kinect object control training. A plan was specifically designed to develop object control skills, and within this plan, children had opportunity to choose the order in which they would play the games. All sessions were led by a single instructor who provided instruction and feedback on how to perform the necessary movements in each game; each session contains warm-up, motor skill instruction and closure activities *CON1*: same lesson structure and content of EXP, but practice was performed in the sports hall and led by a teacher *CON2*: no object control training; outdoor activities at playground area	TGMD-2: Object control skills (striking stationary ball, stationary dribble, kicking, catching, overhand throwing, underhand rolling) Each item received a score of 1 (or 0) if correctly (or not) executed based on standard movement criteria
Ye et al. [35], United States	Non-randomized trial (pre–post), school setting, PE classes	*n* = 261 8.3 ± 0.7 y 51% girls 49% boys	9 months, 2 days/fortnight 25 min/session Unclear total number of sessions	Nintendo Wii; Xbox Kinect. A variety of games were selected from both exergame consoles Participants interacted with the software via a handheld controller and their body movement	*EXP*: participants alternated weeks of regular PE curriculum and weeks of exergaming practice. In the exergaming practice, participants played a variety of exergames individually, in pairs or in group with the supervising teacher or research assistant assisting children in game play throughout *CON*: participants practiced the regular PE curriculum	Maximum kicking speed, maximum throwing speed, maximum long standing jump distance and average hopping height were measured using custom-made tests

*TGMD-2* Test of Gross Motor Development, 2nd edition, *TGMD-3* Test of Gross Motor Development, 3rd edition, *M-ABC-2* Movement Assessment Battery for Children, 2nd edition, *BOT-2* Bruininks–Oseretsky Test of Motor Proficiency, 2nd edition

For each study, the following data were extracted: (a) study information (authors, publication date, country and study design), (b) study sample (sample size, age and sex), (c) the setting in which the experiment took place, (d) the mode of intervention (i.e., technological device or game used), (e) the content of the intervention and who delivered it, (f) the duration of the intervention and (g) FMS measures and outcomes.

Outcome data for the synthesis of results were directly obtained from the included studies, and when data were not fully reported, the corresponding author was contacted (with 100% response rate) to obtain the necessary outcome and effect measure.

### Study Risk of Bias Assessment

The methodological quality of the reviewed studies was evaluated using established risk of bias tools [27]. The risk of bias was assessed using the RoB 2 tool in randomized controlled trials (RCT), an extension of the RoB 2 was used for cluster-randomized trials (RoB 2 Cluster), and the ROBINS-I tool was used for non-randomized trials [28, 29] (tools available at https://www.riskofbias.info/welcome). The RoB 2 tool comprises five bias domains: randomization process, deviations from the intended interventions, missing outcome data, measurement of the outcome and selection of the reported results. The RoB 2 Cluster comprises these same domains with the addition of a bias domain related to identification or recruitment of participants into clusters. The ROBINS-I tool comprises seven bias domains: confounding, selection of participants into the study, classification of interventions, deviations from the intended interventions, missing data, measurement of outcomes and selection of the reported results. All tools contain signaling questions to help assess the potential bias in each domain. In RoB 2 and RoB 2 Cluster tools, there are three possible outcomes in each domain—low, some concerns, and high—while there are five possible outcomes in the ROBINS-I tool—low, moderate, serious, critical and no information. To evaluate and synthesize the studies included in the review parsimoniously, we decided to adjust the rating of ROBINS-I tool to the rating of the RoB 2 tool. As such, low risk remained unchanged, moderate and no information were classified as some concerns, serious and critical were classified as high risk. An overall outcome, corresponding to the highest risk across domains (also known as worst score counts) was calculated for each study (i.e., if the risk was some concerns in one domain only, the overall risk was some concerns). The results of the risk of bias assessment are presented using the traffic light system: green (low), yellow (some concerns) and red (high). Two authors (LO and FS) independently assessed the risk of bias, they cross-checked their results and resolved any discrepancy in a closed meeting, and if consensus was not reached, a third author (JR) was consulted.

### Synthesis Methods

A meta-analytic integration of the results was performed to synthesize the existing literature on the examined hypothesis. For each outcome, the standardized mean difference between experimental and control conditions was used as the effect measure. As such, quantitative FMS outcome measures were extracted from the single studies.

A random effects (RE) model was chosen in order to run a meta-analytic integration of existing results correcting for multiple samples. In order to integrate existing effects from the literature, all effect sizes derived from the single studies were transformed into Pearson correlation coefficients. Within the meta-regression, *r*-to-*z* transformation *escalc* (calculate effect sizes and outcome measures) function in R was used to transform Pearson correlation coefficients into *z* values. A *τ*2 restricted maximum-likelihood estimator was conducted. Results were finally transformed back to Pearson correlation coefficients for better interpretation. All analyses were conducted in R (R Core Team, 2021) with the primary packages *robumeta* [31] and *metafor* [32]. Results of the meta-analytic integration were presented using a forest plot.

## Results

### Study Selection

The search identified 3569 articles, which reduced to 2498 after duplicates were removed. Twenty-nine articles were left after title and abstract screening and were read in full, of which nine articles met the inclusion criteria and were included in this review (Fig. 1).

### Study Characteristics

The characteristics of the included studies are shown in Table 1. The included studies involved 783 participants, with sample sizes ranging from 29 to 261 participants (mean = 87, SD = 69.6, median = 65). The children’s age ranged from 3 to 12 years (median = 8), and the percentage of girls and boys was balanced across the studies (girls, median = 47%). All studies recruited healthy and normally developing participants (i.e., without any psycho-intellectual-physical disability). Mombarg et al. [33] recruited participants with poor balance ability, but without physical or intellectual disability. Two studies sampled populations with low socioeconomic status [34, 35].

Four studies adopted a RCT [33, 36–38], two adopted a cluster-randomized trial [39, 40], and three studies adopted a quasi-experimental design (without randomization) [34, 35, 41]. All studies employed a baseline and post-intervention assessment design, and Vernadakis et al. [38] also included a retention test at a one-month time point. Intervention groups were compared with control groups practicing different activities across studies. The control groups did not engage in any structured practice [33, 34, 36–38], practiced the regular PE curriculum [35, 39, 40] or practiced activities similar to the intervention group but without a technological device [38–41].

Interventions took place in a school-based setting, each carried out at different time points within the school routine: during school time [34, 41], replacing PE classes [35, 38–40], after school [36] or during lunch break [33, 37]. The intervention was supervised by a PE teacher [39–41], motor skill instructor [38], physical therapist [33], research assistant [36] or a combination of PE teacher and research assistants [34, 35]. Johnson et al. [37] did not specify whether and how the intervention was supervised. While in all studies supervision was limited to assisting and motivating children in their game play, Vernadakis et al. [38] used a motor skill instructor for providing specific instructions on how to improve skills. The intervention length differed between one single session and 40 sessions, and the exposure to the technology intervention ranged from a total of 40 min [41] to 800–900 min [34, 40].

All interventions used exergaming as interactive technology. Commercially available exergame stations (e.g., Xbox Kinect and Nintendo Wii) and the provided sport games (e.g., tennis and baseball) were used in all but one study, which used a custom-built exergame station and “goalkeeping” game [41]. In all studies, participants played a variety of activities available from the exergaming games, and in some studies, participants rotated across different exergaming stations [34, 35, 39]. Only Vernadakis et al. [38] implemented a specific and detailed list of game activities with planned progression of tasks for improving FMS.

FMS outcome measures comprised object control skills [34, 36–38, 41], locomotor skills [34], coordination, agility [41], balance and balance-related skills [33, 39, 40]. Object control skills were assessed using the Test of Gross Motor Development 2nd edition (TGMD-2) [42] in Barnett et al. [36] and Vernadakis et al. [38], and using the Test of Gross Motor Development 3rd edition (TGMD-3) [42] in Johnson et al. [37]. Locomotor skills were assessed with the TGMD-2 in Gao et al. [34]. Further, Johnson et al. [37] developed two validated additional skill tests, the golf swing and the putt stroke. Ye et al. [35] assessed object control skills using a custom-made test. Hsiao and Chen [41] measured hand coordination using a custom-made motor competence test. Balance and balance-related skills were assessed using the Movement Assessment Battery for Children 2nd edition (M-ABC-2) [43], the Bruininks-Oseretsky Test of Motor Proficiency 2nd edition (BOT-2) [44], and HUR BT4 platform [45] in Mombarg et al. [33] and Sheehan and Katz [39, 40, 46].

### Risk of Bias in Studies

**Fig. 2:**
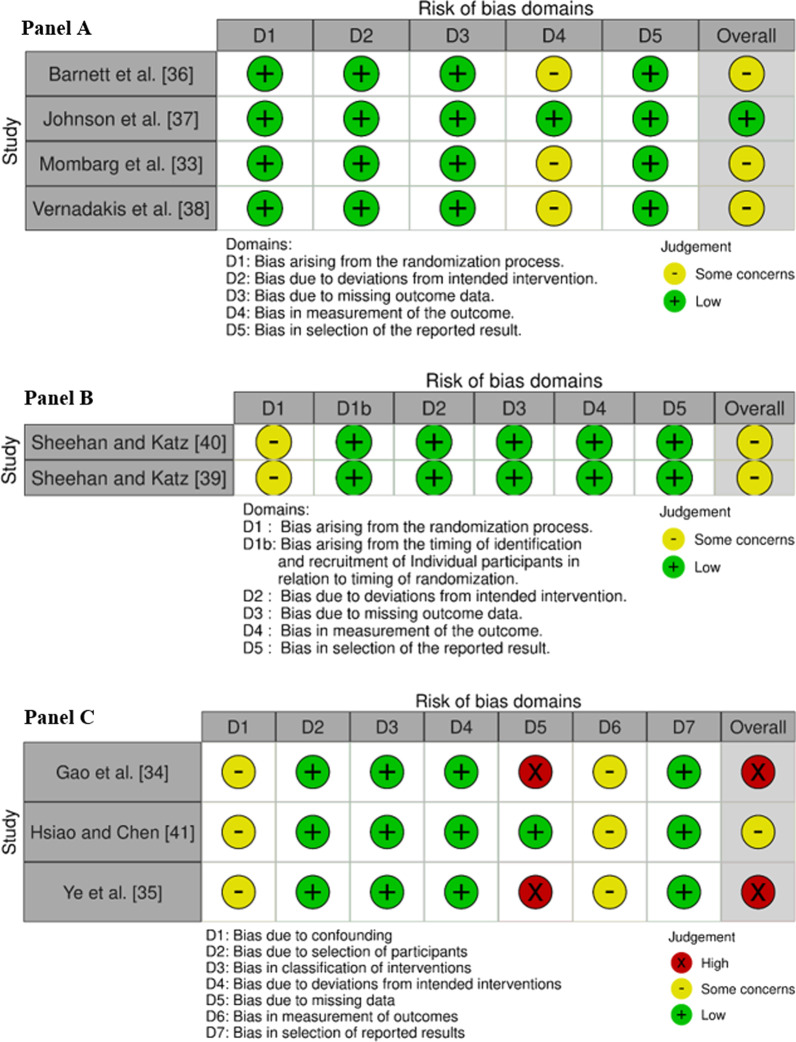
Results of risk of bias assessment for the included studies. RCTs (**A**) were evaluated using the RoB 2 tool, cluster RCTs (**B**) with the RoB 2 Cluster and non-randomized trials (**C**) with the ROBINS-I tool

The risk of bias for the reviewed studies is presented in Fig. 2. One study had a low risk of bias [37], six studies had some concerns [33, 36, 38–41], and two studies had a high risk of bias [34, 35]. Most studies presented concerns at the selection process (bias arising from the randomization process or bias due to confounding: Domain 1). The randomization procedure was poorly reported in the two cluster-randomized trials [39, 40], and some confounding variables were not considered in non-randomized controlled trials [34, 35, 41]. Furthermore, most studies did not report information on blinding of assessors (bias in measurement of the outcome: Domain 4 in RCTs and Domain 6 in non-randomized trials). Lastly, two non-randomized trials [34, 35] poorly reported how they handled missing data, which represented a high risk of bias (bias due to missing data: Domain 5).

### Synthesis of Results

**Fig. 3:**
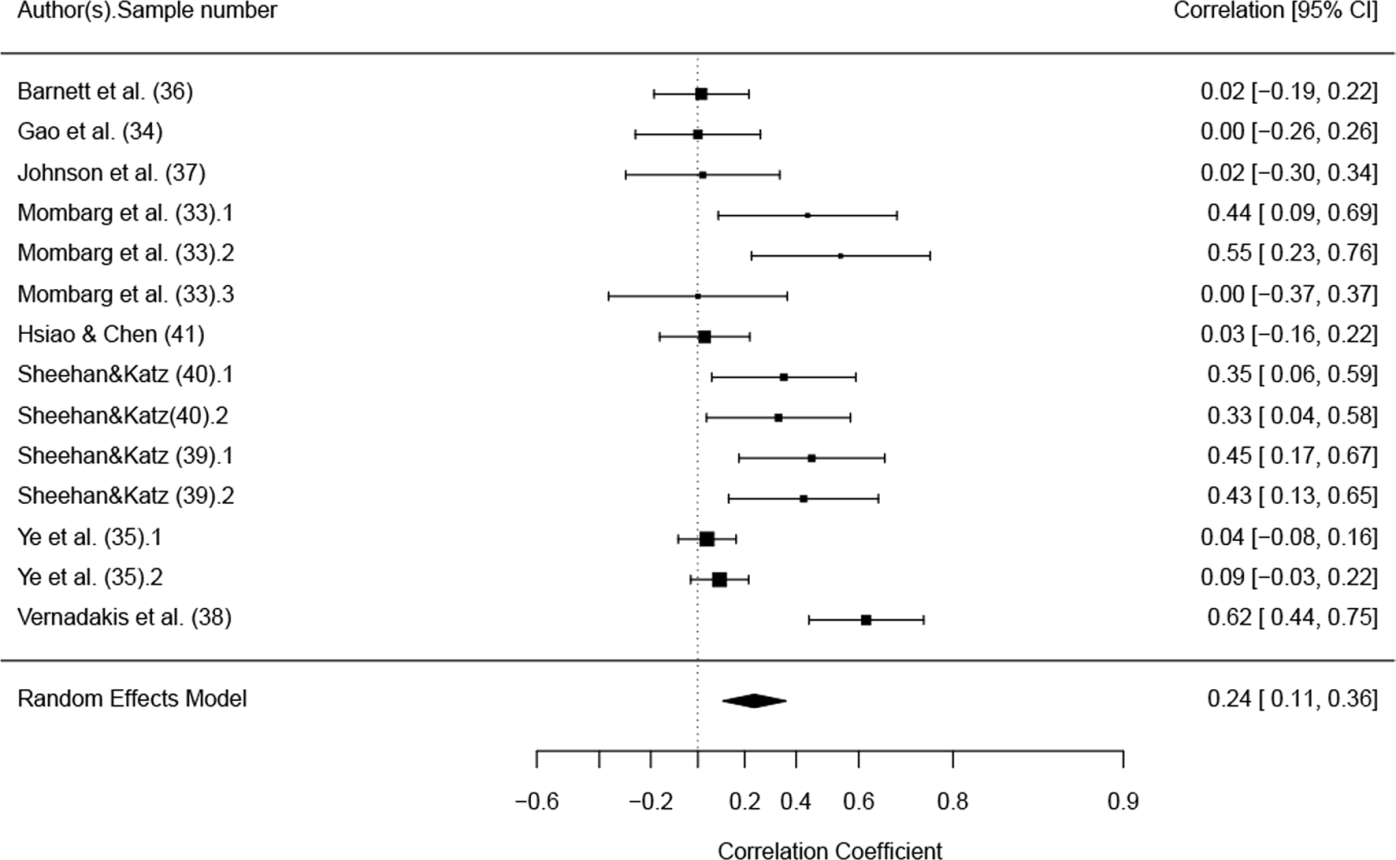
Forest plot showing the differences between the exergaming intervention and control group expressed as correlation coefficient with 95% confidence interval

In order to answer the quantitative research question and summarize the existing evidence within the literature, a meta-analytic integration was performed. The random effects model with multiple sample correction provides evidence toward a small effect of *r* = 0.24 (95% confidence interval 0.11; 0.36) for all studies indicating that a small beneficial effect currently exists in the literature. Results are summarized and presented in Fig. 3.

## Discussion

The aim of this systematic review was to synthesize the evidence on the effectiveness of interventions with immersive and interactive screen-based technology on developing FMS in typically developing children aged three to 12. Nine intervention studies (four RCTs, two cluster-randomized trials and three non-randomized trials) were included in the review. All interventions, except for Barnett et al. [36], took place in a school setting and all used commercially available exergames for the technological intervention, except for a custom-made exergame in Hsiao and Chen [41]. Overall, the meta-analysis revealed small statistically significant intervention effects for FMS development. Due to a low sample size, it was not possible to run a moderator analysis for the type of FMS (object control, locomotor or stability skills). The result of this meta-analysis differs from that of Liu et al. [25] which, without conducting a meta-analysis, concluded that evidence on the effectiveness of active technology (as they called it) on FMS is inconclusive. Further, contrary to Liu et al. [25], we included balance skill as FMS, as current understanding in the movement science considers balance and postural stability to be the foundation for motor learning and control [47, 48].

The benefits of exergames were slightly heterogeneous across studies. The analysis showed that in some of the studies the benefit was null, or in some instances negative (see confidence intervals in Fig. 3), and a close look at the analysis suggests that the positive effect was primarily driven by the studies on balance skills [33, 39, 40] and the Vernadakis et al. [38] study. We interpreted these results in the light of how the games were designed and implemented with respect to the skill under examination (as opposed to limiting the analysis to dose–response relationship, as in Liu et al. [25]). Except for balance, no other trend was detected regarding differential effects across FMS, and we speculate that the benefits of practicing with exergames did not depend on the type of FMS per se but on the specificity of information–movement coupling and the implementation of motor learning strategies. Specificity of information–movement coupling refers to how the information presented in an exergame (e.g., a flying ball) and the movement coupled with that information (e.g., a catching or hitting movement) are specific to the behavior targeted in the task (object interception, e.g., catching a ball thrown by another person or hitting a forehand in tennis) [49]. Common strategies for promoting motor learning are instruction, augmented feedback and manipulation of task difficulty [50, 51].

Commercially available balance-related exergames, such as Wii Fit Plus and snowboard simulators, are designed for improving balance and contain motor learning strategies and a high specificity of information–movement coupling. When interacting with the software, children stand on a platform and continuously control their balance using augmented visual feedback displayed on a monitor about their center of gravity in relation to the task environment. Augmented feedback is key for promoting motor learning, as it guides a learner in their search for stable and effective movement solutions [52], and such a strategy is typically employed for teaching or re-teaching, balance (e.g., Mansfield et al. [53]). Further, the platform and software design provide a high specificity of information (about a child’s center of gravity) and movement coupling (a child’s movement and displacement of center of gravity are accurately reflected in the visual display). It is likely that this coupling facilitates two key processes of movement learning: first, attunement of perception to task-relevant information (e.g., center of gravity in relation to one’s own base of support) and secondly, calibration of action (perceptual information is calibrated into movement units) [54]. Why high task specificity is important here is that it promotes transfer to the “real-world” context of skill performance [55]. Furthermore, the game software typically contains other important skill acquisition principles such as options to change difficulty, different scenarios and challenges, and avatar development all of which can support a child’s improvement of their balance and keep a child motivated to continue in the game.

Commercially available exergames that include object control and locomotor activities typically have a low specificity of information–movement coupling and do not contain skill-specific motor learning strategies. The sensor technology embedded in exergames has limitations in tracking movement accurately, especially in whole-body dynamic movements, and the link between a user’s movement and information on the screen is quite approximate and thus far from a skill employed in the “real world.” For example, a child can play a tennis game just by moving their wrist, and these movements will be represented as full arm swing on the display. This low coupling specificity will likely transfer poorly to playing tennis at the local park. We speculate that this low specificity in commercially available exergames, which is due to the inaccuracy of the tracking system, means that they do not promote the development of object control and locomotor skills. This also explains why the effect size found in this review is smaller than those observed in a previous meta-analysis for FMS interventions that took place in the “real world” [56]. Vernadakis et al. [38], however, demonstrate that if current exergame technology is supplemented with motor learning principles, implemented instruction and augmented feedback tailored to each individual child (guiding them to improve their movement) with children modulating the task difficulty of the chosen game, then these strategies will lead to FMS improvement similar to those seen in PE lessons.

The need for exergames to be principled in motor learning theories for promoting the development of FMS is further supported in the literature [57]. McGann et al. [58] compared commercial exergame technology with a purpose-built exergame that was designed to improve children’s FMS (locomotor skills) utilizing a principled approach to skill acquisition (assessment, rules, challenge, feedback and instruction). Significant improvement in all FMS was observed when compared to commercial-based technology [58]. While it was not possible to include this study in our systematic review, as it did not have a control group, its results highlight the potential of screen-based exergames to improve movement skills, and indeed, these may surpass what is possible in PE alone. This should not be seen as a recommendation for exergaming to replace PE but rather that a screen-based technology that can benefit children’s educational and health outcomes, and is complementary to PE, could have an impact in improving FMS globally, reducing the amount of harmful sedentary-based screen time [12, 59].

Future research should seek to improve the sensitivity and accuracy of exergaming sensor technology as well as the representative design of the game so that it mirrors “real-world” performance contexts. Exergames that are developed in this manner will result in specifying information that is highly representative to real-world context and therefore increase the likelihood that FMS developed in the game transfer to a multitude of physical activity environments in the real world. This provides new insights that FMS improvements are not simply determined by the type of FMS practiced or the total amount of practice but are also due to the quality of the specifying information to which the user couples. Further, physical educators should be trained to support children’s FMS development using a principled approach to skill acquisition. In future, recommendations for children’s screen time may include consideration of the type of screen time that children are engaging in, rather than giving a blanket recommendation to limit its amount, which is in our opinion unlikely to be realistic in today’s techno-proliferation.

This review has a number of strengths: (1) a comprehensive search strategy across multiple databases to detect the full range of relevant studies, (2) extensive study detail extracted with broad inclusion criteria and (3) alignment with the PRISMA Statement which provides transparency and rigor on the conducted meta-analysis procedure. There were also a number of limitations. Studies were required to be published in English, which may have excluded studies published in other languages; the meta-analysis included a generally modest number of heterogeneous studies, which may reduce the precision of the effect size estimate and may not provide the full picture on exergaming effectiveness; inability to conduct a moderator analysis on the type of activities control groups performed and on the different types of FMS due to low sample size, and consequently difficulties in comparing the effect of different studies. This last point is of particular relevance, as exergaming may have a differential effect on different FMS. For instance, a close look at Fig. 3 may suggest that exergaming is mostly beneficial for balance and postural skills, i.e., Mombarg et al. [33], and Sheehan and Katz [39, 40] showed an enhanced improvement in balance skills with the exergaming intervention relative to control. Future research is required to further examine this issue, as well as to explore whether exergaming is more beneficial for children with low FMS as was the case in the Mombarg et al. [33] study.

## Conclusion

Our review showed a small benefit of using exergames for improving FMS in children. The benefits are likely to be dependent on exergame design with respect to the target FMS and the strategies implemented during an intervention. Exergames are designed for encouraging children to exercise; however, they improve FMS only where activities are specifically designed for improving FMS. Existing balance games (e.g., Wii Fit Plus) contain elements for improving balance, but other games do not currently contain elements for improving FMS, such as object control skills. In view of this, it is recommended that interventions should incorporate skill acquisition principles and theories of motor learning to help improve FMS and that games should be designed specifically to improve FMS. It would be interesting to examine how recent development in gaming technology (e.g., headset virtual reality) may facilitate the implementation of the discussed motor learning principles. Ultimately, this study provides new insights that can shift the current view on screen-based technology, suggesting that such technology can be beneficial for promoting children’s FMS development, and practitioners are encouraged to explore its implementation in their practice.

## Data Availability

Not applicable.

## Abbreviations

FMS: Foundational movement skills
PE: Physical education
RCT: Randomized controlled trial
RE: Random effect
TGMD-2: Test of Gross Motor Development 2nd edition
TGMD-3: Test of Gross Motor Development 3rd edition
M-ABC-2: Movement Assessment Battery for Children 2nd edition
BOT-2: Bruininks-Oseretsky Test of Motor Proficiency 2nd edition
